# Impact of the COVID-19 era on preventative primary care for children 0–5 years old: a scoping review protocol

**DOI:** 10.1186/s13643-024-02507-2

**Published:** 2024-04-26

**Authors:** Xin Qi, Mackenzie Jordan, Isabella Mignacca, Imaan Bayoumi, Patricia Li

**Affiliations:** 1 School of Medicine, Queen’s University, Kingston, ON Canada; 2 Royal College of Surgeons in Ireland, Dublin, Ireland; 3 Department of Family Medicine, McGill University, Montréal, QC Canada; 4 Department of Family Medicine, School of Medicine, Queen’s University, Kingston, ON Canada; 5 Department of Pediatrics, Faculty of Medicine and Health Sciences, Research Institute of the McGill University Health Centre, McGill University, Montréal, QC Canada

**Keywords:** COVID-19, Preventative medicine, Primary care, Paediatric, Well-child care, Well-baby care

## Abstract

**Background:**

The COVID-19 pandemic has resulted in widespread disruptions to primary healthcare delivery and shifts to virtual care. Reduced in-person paediatric primary care visit rates have been reported. However, the extent to which access to primary preventative care has been impacted remains unclear. The objective of this scoping review is to characterise the utilisation of preventative primary care and its association with child development for children ages 0–5 years old during the COVID-19 era. In addition, we will determine if specific groups of children are at greater risk for reduced access to care.

**Methods:**

A systematic search will be conducted for studies published between March 11, 2020, and October 2023 in the following databases: MEDLINE (Ovid), Embase (Ovid), Cochrane Library (CENTRAL and CDSR), Web of Science, and CINAHL (EBSCOhost). This scoping review will follow the methodological framework developed by Arksey and O’Malley and updated by the Joanna Briggs Institute (JBI). Studies related to primary preventative care of children aged 0–5 years old conducted in English and in high-income countries will be screened. Studies published before March 11, 2020, in acute care and low–middle-income settings will be excluded. Results will be summarised for appointments attended, delayed, and missed. In addition, we will summarise findings on the impact of COVID-19 on child development. Findings will be reported according to the Preferred Reporting Items for Systematic Reviews and Meta-Analyses Extension for Scoping Reviews (PRISMA-ScR) guidelines.

**Discussion:**

Further investigation is required to better understand the relationship between attendance of preventative primary care for children and its effects on child development. The findings obtained from this review will offer essential context to guide policy-making and healthcare service planning for the period following the pandemic.

## Background

During the COVID-19 pandemic, widespread disruptions to primary health care delivery resulted in shifts from primary care settings, defined as care delivered in clinics, community health centres, ambulatory settings, urgent care, schools, and at-home visits, to virtual and mobile care. The COVID-19 pandemic has impacted preventative primary health care delivery [1–4]. but the extent to which it has affected access to preventative primary care for young children (0–5 years old) remains unclear. For example, virtual care became an important means for the delivery of primary care but may not be ideal for children where monitoring of growth and identification of developmental delays are better assessed using in-person visits [5].

Preventative primary care provided in early childhood is essential for the prevention of future health problems [6, 7]. Early childhood (between the ages of 0–5 years old) is marked by rapid growth and brain development, making it a sensitive period of life where children are particularly vulnerable to stressors, such as disruptions to routine, socialisation, and education that result from the COVID-19 pandemic [8]. There is evidence that in-person paediatric primary care visit rates declined in the USA during the COVID-19 pandemic [9] and that the pandemic had the potential to impact infant and toddler development [10]. Implications between the decline of preventative primary care in the paediatric population and child development need to be further clarified. As such, we propose to conduct a scoping review on the impact of the COVID-19 era (March 11, 2020-present) on preventative primary care and child development outcomes in children 0–5 years old. Insights gained from this review will provide the background necessary to inform policy and health services planning for the post-pandemic era and future pandemics.

## Methods/design

This protocol is for a scoping review of the literature reporting on the impact of the COVID-19 era on preventative primary care and developmental assessment for children ages 0–5 years old in high-income countries (HIC). A scoping review method was chosen to evaluate the extent and range of knowledge contributed to the field thus far and to identify gaps in the current literature to prompt further research. The scoping review will be guided by the methodological framework proposed by Arksey and O’Malley in 2005 and updated by the Joanna Briggs Institute (JBI) in 2020 [11, 12]. The five stages specified by Arksey and O’Malley will be followed: (1) identifying the research question, (2) identifying relevant studies, (3) study selection, (4) charting the data, and (5) collating, summarising, and reporting the results. Quality appraisal will not be formally assessed as recommended by JBI scoping review guidelines because the goal is to evaluate the extent of available evidence in this field [12].

### Stage 1: identifying the research question

The main research question is as follows: what is the impact of the COVID-19 era (March 11th, 2020–present) on preventative primary care visits and developmental outcomes for children aged 0–5 years old? Specifically,

1. What were the changes in attendance for in-person and virtual visits provided by primary care providers in primary care settings?
2. What is the impact of the COVID-19 era on child development?
3. How did health services disruptions in primary care impact the diagnosis and management of developmental delays in children 0–5?
4. Are there specific groups of children who experienced more challenges in accessing primary preventative care and how did this impact the diagnosis and management of developmental delays?

This study will use the population-concept-context (PCC) framework suggested by the JBI scoping review guidelines to help highlight the key concepts of the main research question, as presented in Table 1 [12].

**Table 1:** Population-concept-context

Population	Healthy children between the ages of 0–5 years old will be included. Children born premature or living with complex chronic conditions will be excluded from this review, as well as research conducted outside of high-income countries.
Concept	Literature reporting on the frequency/rate and main reasons for preventative primary care visit delays during the COVID-19 pandemic will be reviewed. Literature reporting on outcomes related to developmental concerns (such as speech/language delay and identification of autism spectrum disorder) will be included.
Context	The time frame is from the start of the COVID-19 pandemic (March 11, 2020) onwards. The context will be focused on primary care settings as defined as care delivered in clinics, community health centres, ambulatory settings, virtual care, urgent care, mobile clinics, schools, and at-home visits. Primary care providers include family physicians, general paediatricians, nurse practitioners, physician assistants, and registered nurses. We will be focusing on access to preventative care and on the impacts of identifying developmental concerns. Given the diversity of primary care systems across the globe, we will attempt to minimise heterogeneity by examining research conducted in high-income countries only.

### Stage 2: identifying relevant studies

Our search strategy and database selection were supported by trained medical librarians. A comprehensive search was conducted in October 2023 for studies published between March 11, 2020–October 2023 in the following online databases: MEDLINE (Ovid), Embase (Ovid), Cochrane Library (CENTRAL and CDSR), Web of Science, and CINAHL (EBSCOhost). Key search terms were developed to identify studies related to the impact of the COVID-19 pandemic on paediatric preventative primary care visits, as well as their impact on child developmental outcomes. The search strategy involved studies published as of March 11, 2020, with related Medical Subject Headings (MeSH) terms or keywords such as “COVID-19”, “primary health care”, “paediatric” or “well child visit”, and “child development”. A filter for COVID-19 constructed by the University of Alberta Health Sciences Library was used to search for COVID-19 and related concepts [13]. The search strategy was piloted and refined to check the appropriateness of articles pertaining to the main concept. The search strategy was adapted for each included database. The finalised search strategy used to search in MEDLINE (Ovid) is available in Appendix 1.

### Stage 3: study selection

Title and abstract screening will be guided by the PCC framework (Table 1). Further eligibility criteria outlined below will ensure that the context and content of the studies found are relevant to the research questions.

#### Inclusion criteria

- Children aged 0–5 years old. For studies that include a wider range of ages not limited to 0–5 years old (for example, children 0-18 years old), we will apply the following inclusion criteria: (a) if the mean or median age of children is 5 years old or less, or if the distribution of children includes at least 50% children 0–5 years old; and (b) consider only the data for children 0–5 years old if a study reports outcomes by age or age categories.
- Studies published during the COVID-19 era (between March 11, 2020 and October 2023).
- Related to primary preventative care, access to primary preventative care, or child development outcomes.
- Research completed in high-income countries (HIC).
- Study design: Experimental and quasi-experimental study designs including qualitative and quantitative studies, literature, systematic, and scoping reviews, randomised controlled trials, non-randomised controlled trials, interrupted time-series studies, descriptive and analytical observational studies, including prospective and retrospective cohort studies, case-control studies, and cross-sectional studies.

#### Exclusion criteria

- Children 6 years old or older, born premature, or living with complex chronic conditions. For studies with an age range beyond 0–5 years old, we will exclude those that do not report either the mean age, median age, or the outcomes by age or age categories.
- Studies published before the COVID-19 era (before March 11th, 2020).
- Non-primary care settings (e.g., acute care provided in the emergency room or hospitals).
- Work completed in low- and middle-income countries.
- Study design: exclude case reports and series, opinion pieces, commentaries, editorials, abstracts, conference proceedings, grey literature, and non-English studies.

Following the search, all identified citations will be collated and uploaded into Covidence and duplicates removed. After the initial pilot test, titles and abstracts will be screened by two independent reviewers to assess whether the citation meets the inclusion criteria. Those determined to be potentially relevant sources will be retrieved in full text. The full text of selected citations will be assessed in detail against the inclusion criteria by two independent reviewers. Reasons for exclusion of sources of evidence in full text that do not meet the inclusion criteria will be recorded and reported in the scoping review. Any disagreements that arise between the reviewers at each stage of the selection process will be resolved through discussion, or with an additional reviewer/s. The results of the search and the study inclusion process will be reported in full in the final scoping review and presented in a Preferred Reporting Items for Systematic Reviews and Meta-analyses extension for scoping review (PRISMA-ScR) flow diagram [14].

### Stage 4: charting the data

Data will be extracted from papers included in the scoping review by two independent reviewers using a data extraction form developed by the reviewers. The data extracted will include specific details about the study participants, concept, context, study methods, and key findings relevant to the review questions. A draft data extraction form will initially be pilot-tested using 3–5 papers and modified to address feedback from reviewers prior to implementation of the form for all relevant articles through an iterative process.

Extracted data will include the following: (1) bibliometric details: Title, author(s), and country in which the study was conducted; (2) study characteristics: study aims, design, start date, end date, funding sources, conflicts of interest, age of study participants, description, inclusion and exclusion criteria, method of recruitment, sample size, and study sample characteristics including age, race, and ethnicity; (3) study outcomes: changes in visit and referral rates reported before and during the COVID-19 pandemic, including frequencies of in-person, phone, and video visits, and frequency of delayed and missed appointments. The impact of the COVID-19 era on changes in child development will be extracted by summarising the key findings from each paper relevant to our objectives.

### Stage 5: collating, summarising, and reporting the results

The PRISMA Extension for Scoping Reviews (PRISMA-ScR) checklist will be followed to report the results of this scoping review [14]. To answer objectives (1) and (2), results of the scoping review will be described using frequency counts of changes in attendance for in-person and virtual visits, and delays in referral times for specialist appointments. This will be presented in a table, while bibliometric details about the populations, samples, and location of studies will be reported according to standard scoping review methodological reporting guidelines. To answer objectives (3) and (4), extracted data will highlight and summarise trends about the impact of the COVID-19 era on child development and the extent to which barriers in accessing primary care impacted the diagnosis of developmental delays. These summaries will be reported narratively. Gaps in the literature will also be identified.

## Discussion

The proposed scoping review uses a well-established framework to provide an overview of available evidence and to identify gaps in the literature related to preventative primary care for young children during the COVID-19 pandemic. Upon completion of this review, we will be able to summarise the existing literature regarding the impact of COVID-19 on primary care visit rates and developmental outcomes in children aged 0–5 years. We will also be able to make recommendations for future research based on areas of interest related to paediatric primary care delivery during pandemic conditions that are currently lacking evidence. Additionally, this scoping review will be foundational in providing context to guide policymakers and suggest best practices in the event of another global health crisis, such as a pandemic.

## Data Availability

Not applicable.

## Appendix 1: Search strategies

### Search Strategy for MEDLINE (Ovid) and Cochrane Library (CENTRAL and CDSR)

Ovid MEDLINE(R) ALL <1946 to October 23, 2023>

1. (((exp Coronavirus/ or exp Coronavirus Infections/ or (coronavirus* or corona virus* or OC43 or NL63 or 229E or HKU1 or HCoV* or ncov* or covid* or sars-cov* or sarscov* or Sars-coronavirus* or Severe Acute Respiratory Syndrome Coronavirus*).mp.) and (201906* or 201907* or 201908* or 201909* or 20191* or 2020* or 2021* or 2022* 2023* or 2024* or 2025* or 2026* or 2027* or 2028* or 2029* or 2030*).dt,ez,da.) not (SARS or SARS-CoV or MERS or MERS-CoV or Middle East respiratory syndrome or camel* or dromedar* or equine or coronary or coronal or covidence* or covidien or influenza virus or HIV or bovine or calves or TGEV or feline or porcine or BCoV or PED or PEDV or PDCoV or FIPV or FCoV or SADS-CoV or canine or CCov or zoonotic or avian influenza or H1N1 or H5N1 or H5N6 or IBV or murine corona*).mp.) or (Covid-19/ or covid.mp. or covid19.mp. or 2019-ncov.mp. or ncov19.mp. or ncov-19.mp. or 2019-novel CoV.mp. or sars-cov2.mp. or sars-cov-2.mp. or sarscov2.mp. or sarscov-2.mp. or Sars-coronavirus2.mp. or Sars-coronavirus-2.mp. or SARS-like coronavirus*.mp. or coronavirus-19.mp. or Deltacron.mp. or Omnicron.mp. or ((novel or new or nouveau) adj2 (CoV or nCoV or covid or coronavirus* or corona virus or Pandemi*2)).mp. or ((subvariant* or variant*) adj2 (India* or "South Africa*" or UK or English or Brazil* or alpha or beta or delta or gamma or kappa or lambda or mu or "AY.X" or "BA.1" or "BA.2" or "BA.3" or "BA.4" or "BA.5" or "P.1" or "C.37")).mp. or ("B.1.1.7" or "B.1.351" or "B.1.617.1" or "B.1.617.2" or "B.1.1.529*" or "B.1.61.7*" or "21L/BA.2" or "21K/BA.1").mp.) [mp=title, book title, abstract, original title, name of substance word, subject heading word, floating sub-heading word, keyword heading word, organism supplementary concept word, protocol supplementary concept word, rare disease supplementary concept word, unique identifier, synonyms]
2. exp Preventive Medicine/
3. exp Primary Health Care/
4. exp "Referral and Consultation"/
5. exp "Appointments and Schedules"/
6. exp Telemedicine/
7. Child Health Services/
8. (preventative adj2 care).mp. [mp=title, book title, abstract, original title, name of substance word, subject heading word, floating sub-heading word, keyword heading word, organism supplementary concept word, protocol supplementary concept word, rare disease supplementary concept word, unique identifier, synonyms]
9. routine appointment*.mp.
10. in person visit*.mp.
11. in-person visit*.mp.
12. virtual visit*.mp.
13. well baby care.mp.
14. well baby visit*.mp.
15. well child care.mp.
16. well child visit*.mp.
17. 2 or 3 or 4 or 5 or 6 or 7 or 8 or 9 or 10 or 11 or 12 or 13 or 14 or 15 or 16
18. child, preschool/ or exp infant/
19. Pediatrics/
20. child*.mp.
21. infant*.mp.
22. baby.mp.
23. babies.mp.
24. toddler*.mp.
25. paediatric*.mp.
26. 18 or 19 or 20 or 21 or 22 or 23 or 24 or 25
27. exp child development/ or exp language development/
28. (speech and language).mp. [mp=title, book title, abstract, original title, name of substance word, subject heading word, floating sub-heading word, keyword heading word, organism supplementary concept word, protocol supplementary concept word, rare disease supplementary concept word, unique identifier, synonyms]
29. exp autism spectrum disorder/ or exp asperger syndrome/ or exp autistic disorder/
30. exp child behavior disorders/ or exp child development disorders, pervasive/ or exp communication disorders/ or exp developmental disabilities/
31. 27 or 28 or 29 or 30
32. 17 or 31
33. 1 and 26 and 32

### Search Strategy for Embase (Ovid)

Embase Classic+Embase <1947 to 2023 October 23>

1. ((exp Coronavirus/ or exp Coronavirus Infections/ or (coronavirus* or corona virus* or OC43 or NL63 or 229E or HKU1 or HCoV* or ncov* or covid* or sars-cov* or sarscov* or Sars-coronavirus* or Severe Acute Respiratory Syndrome Coronavirus* or D614G).mp.) not (SARS or SARS-CoV or MERS or MERS-CoV or Middle East respiratory syndrome or camel* or dromedar* or equine or coronary or coronal or covidence* or covidien or influenza virus or HIV or bovine or calves or TGEV or feline or porcine or BCoV or PED or PEDV or PDCoV or FIPV or FCoV or SADS-CoV or canine or CCov or zoonotic or avian influenza or H1N1 or H5N1 or H5N6 or IBV or murine corona*).mp.) or coronavirus disease 2019/ or ((exp pneumonia/ or (pneumonia or covid* or coronavirus* or corona virus* or ncov* or 2019-ncov or sars*).mp.) and Wuhan.mp.) or ("coronavirus disease 2019" or 2019-ncov or ncov19 or ncov-19 or 2019-novel CoV or severe acute respiratory syndrome coronavirus 2 or sars-cov2 or sars-cov-2 or sarscov2 or sarscov-2 or Sars-coronavirus2 or Sars-coronavirus-2 or SARS-like coronavirus* or coronavirus-19 or covid19 or covid-19 or "covid 2019" or "B.1.1.7" or "B.1.351" or "B.1.617.1" or "B.1.617.2" or omicron or Deltacron).mp. or ((subvariant* or variant*) adj2 (India* or "South Africa*" or UK or English or Brazil* or alpha or beta or delta or gamma or kappa or lambda or mu or "AY.X" or "BA.1" or "BA.2" or "BA.3" or "BA.4" or "BA.5" or "P.1" or "C.37")).mp. or ("B.1.1.7" or "B.1.351" or "B.1.617.1" or "B.1.617.2" or "B.1.1.529*" or "B.1.61.7*" or "21L/BA.2" or "21K/BA.1").mp. or ((novel or new or nouveau) adj2 (CoV or nCoV or coronavirus* or corona virus)).mp. [mp=title, abstract, heading word, drug trade name, original title, device manufacturer, drug manufacturer, device trade name, keyword heading word, floating subheading word, candidate term word]
2. exp preventive medicine/
3. exp Primary Health Care/
4. exp patient referral/
5. exp consultation/
6. exp Telemedicine/
7. exp child health care/
8. (preventative adj2 care).mp. [mp=title, abstract, heading word, drug trade name, original title, device manufacturer, drug manufacturer, device trade name, keyword heading word, floating subheading word, candidate term word]
9. routine appointment*.mp.
10. in person visit*.mp.
11. in-person visit*.mp.
12. virtual visit*.mp.
13. well baby care.mp.
14. well baby visit*.mp.
15. well child care.mp.
16. well child visit*.mp.
17. 2 or 3 or 4 or 5 or 6 or 7 or 8 or 9 or 10 or 11 or 12 or 13 or 14 or 15 or 16
18. exp child/ or exp preschool/ or exp infant/
19. Pediatrics/
20. child*.mp.
21. infant*.mp.
22. baby.mp.
23. babies.mp.
24. toddler*.mp.
25. paediatric*.mp.
26. 18 or 19 or 20 or 21 or 22 or 23 or 24 or 25
27. exp child development/ or exp language development/
28. (speech and language).mp. [mp=title, abstract, heading word, drug trade name, original title, device manufacturer, drug manufacturer, device trade name, keyword heading word, floating subheading word, candidate term word]
29. exp autism/ or exp asperger syndrome/
30. exp behavior disorder/ or exp attention deficit hyperactivity disorder/ or exp externalizing disorder/ or exp internalising disorder/
31. 27 or 28 or 29 or 30
32. 17 or 31
33. 1 and 26 and 32

### Search Strategy for Web of Science

((ALL=(COVID-19 OR coronavirus OR “coronavirus infection*” OR sars-cov* OR sars-coronavirus* OR “Severe Acute Respiratory Syndrome Coronavirus*”)) AND ALL=("preventative medicine" OR "primary health care" OR "referral and consultation" OR appointments OR schedules OR telemedicine OR "child health services" OR "preventative health care" OR "routine appointment" OR "in person visit" OR "in-person visit" OR "virtual visit" OR "well baby care" OR "well baby visit*" OR "well child care" OR "well child visit*" OR “child development” OR “language development” OR “speech and language” OR autism OR “asperger syndrome” OR “autistic disorder” OR “child behavior disorder*” OR “child development disorder*” OR “communication disorder*” OR “developmental disabilities”)) AND ALL=(child* OR preschool OR infant* OR pediatric* OR paediatric* OR baby OR babies OR toddler* )

### Search Strategy for CINAHL (EBSCOhost)

((MH "COVID-19") OR (MH "SARS-CoV-2") OR (MH "COVID-19 Pandemic") OR (MH "Coronavirus Infections") OR (MH "Coronavirus")) AND (((MH "Child") OR (MH "Infant+") OR (MH "Child, Preschool")) OR "pediatric" OR child OR infant OR baby OR babies OR toddler OR paediatric) AND ((((MH "Preventive Health Care+")) OR ((MH "Primary Health Care")) OR ((MH "Referral and Consultation") OR (MH "Remote Consultation")) OR ((MH "Appointments and Schedules+")) OR ((MH "Telemedicine+")) OR ((MH "Child Health Services+")) OR ("preventative care") OR ("routine appointment") OR ("in person visit") OR (in-person visit) OR (virtual visit) OR (well baby care) OR (well baby visit) OR (well child care) OR (well child visit)) OR (((MH "Child Development") OR (MH "Language Development")) OR (speech AND language) OR ((MH "Autistic Disorder") OR (MH "Asperger Syndrome")) OR ((MH "Child Behavior Disorders") OR (MH "Child Development Disorders") OR (MH "Infant Development Disorders") OR (MH "Communicative Disorders") OR (MH "Developmental Disabilities"))))

## References

[1] Who director-general's opening remarks at the media briefing on COVID-19 - 11 March 2020. World Health Organization. 2020.https://www.who.int/director-general/speeches/detail/who-director-general-s-opening-remarks-at-the-media-briefing-on-covid-19---11-march-2020. Accessed 4 July 2022.

[2] Covid-19 collection: Primary care covid-19 survey: Larry Green Center and Primary Care Collaborative. Annals of Family Medicine. 2020.https://www.annfammed.org/content/covid-19-collection-primary-care-covid-19-survey-larry-green-center-and-primary-care. Accessed 4 July 2022.

[3] Glazier RH, Green ME, Wu FC, et al. Shifts in office and virtual primary care during the early COVID-19 pandemic in Ontario, Canada. Can Med Assoc 2021;193.https://www.cmaj.ca/content/193/6/E200. Accessed 4 July 2022. 10.1503/cmaj.202303 PMC7954541 33558406

[4] Schweiberger K,  Patel SY, Mehrotra  A, Ray   KN (2021). Trends in pediatric primary care visits during the coronavirus disease of 2019 pandemic. Acad Pediatr.

[5] Tully L, Case L, Arthurs N, Sorensen J, Marcin JP, O'Malley G (2021). Barriers and facilitators for implementing paediatric telemedicine: rapid review of user perspectives. Frontiers in pediatrics.

[6] Yanchar NL, Warda LJ, Fuselli P (2012). Child and youth injury prevetion: a public health approach. Paediatr Child Health.

[7] Starfield B, Shi L,  Macinko J (2005). Contribution of primary care to health systems and health. Milbank Q.

[8] Bhutta ZA, Guerrant RL, Nelson CA. Neurodevelopment, nutrition, and inflammation: The Evolving Global Child Health Landscape. Pediatrics. 2017;139(Suppl 1):S12–22. https://publications.aap.org/pediatrics/article-abstract/139/Supplement_1/S12/34124/Neurodevelopment-Nutrition-and-Inflammation-The?redirectedFrom=fulltext. Accessed 4 July 2022. 10.1542/peds.2016-2828D 28562245

[9] Lebrun-Harris LA, Sappenfield OR, Warren MD (2021). Missed and delayed preventive health care visits among us children due to the COVID-19 pandemic. Public Health Reports..

[10] Imboden A, Sobczak BK, Griffin V (2021). The impact of the COVID-19 pandemic on Infant and Toddler Development. J Am Assoc Nurse Pract..

[11] Arksey H, O'Malley L (2007). Scoping studies: Towards a methodological framework. Int J Soc Res Methodol..

[12] Peters M, Godfrey C, McInerney P, et al. Chapter 11: Scoping reviews. JBI Manual for Evidence Synthesis Published Online First: 2020. https://jbi-global-wiki.refined.site/space/MANUAL/4687833/11.1+Introduction+to+Scoping+reviews. Accessed 31 July 2022.

[13] Campbell S. Modified versions of Wolters Kluwer Covid... ERA. 2020.https://era.library.ualberta.ca/items/bb8b4739-571c-43de-a690-edb192ab468f. Accessed 3 July 2022.

[14] Moher D, Liberati A, Tetzlaff J, et al. Preferred reporting items for systematic reviews and meta-analyses: The Prisma statement. BMJ 2009;339.https://www.bmj.com/content/339/bmj.b2535. Accessed 3 July 2022. PMC3090117 21603045

